# Supplementary material for: Quantifying the Patterns of Metabolic Plasticity and Heterogeneity along the Epithelial–Hybrid–Mesenchymal Spectrum in Cancer
Source: Biomolecules. 2022 Feb 12;12(2):297. doi: 10.3390/biom12020297 (PMC8961667; doi:10.3390/biom12020297)
Supplement: Supplementary file 1 [file biomolecules-12-00297-s001.zip › biomolecules-1538640-final-sup.pdf]

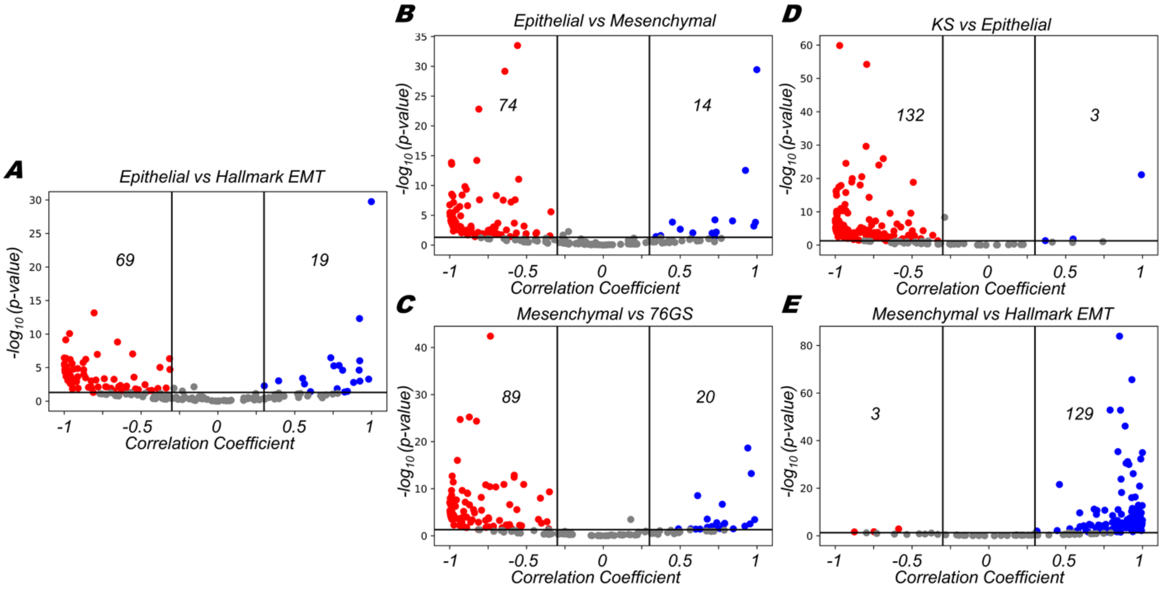

Figure S1. Consistency between different EMT scoring metrics. Volcano plots depicting the Pearson correlation coefficient and the −log10(*p*-value) for (**A**) Epithelial vs Hallmark EMT (**B**) Epithelial vs Mesenchymal (**C**) Mesenchymal vs 76GS (**D**) KS vs Epithelial (**E**) Mesenchymal vs Hallmark EMT. Vertical boundaries are set at correlation coefficient −0.3 and 0.3. The cut-off for p-value is set at 0.05.

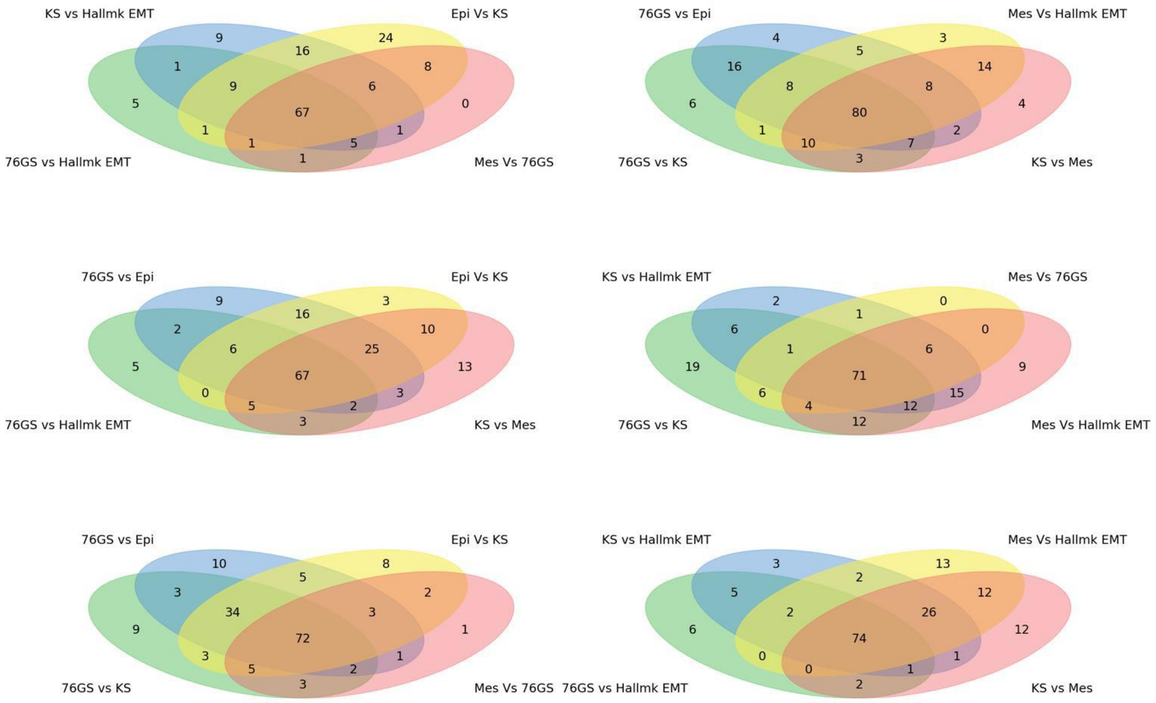

Figure S2. Different pairs of EMT scoring metrics show a strong overlap across multiple datasets that have expected trends. Representative 4-way Venn diagrams are depicted for comparison between different EMT metrics.

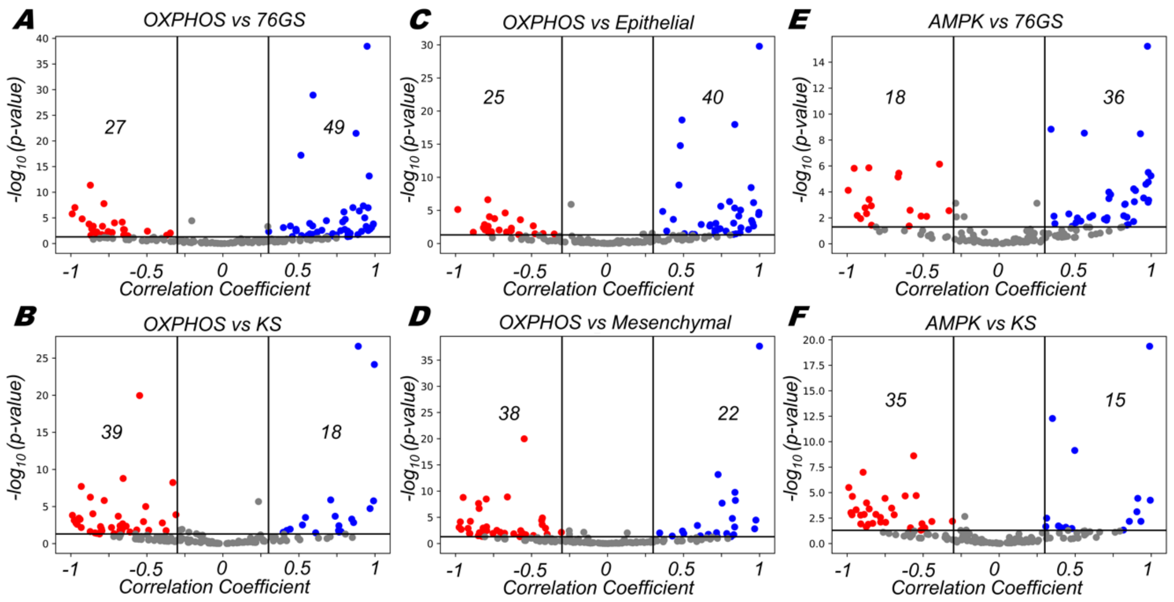

Figure S3. OXPHOS and its regulator AMPK is more likely to correlate negatively with EMT. Volcano plots depicting the Pearson correlation coefficient and the −log10(p-value) for (**A**) OXPHOS vs 76GS (**B**) OXPHOS vs KS (**C**) OXPHOS vs Epithelial (**D**) OXPHOS vs Mesenchymal (**E**) AMPK vs 76GS (**F**) AMPK vs KS. Vertical boundaries are set at correlation coefficient −0.3 and 0.3. Cut-off for p-value is set at 0.05.

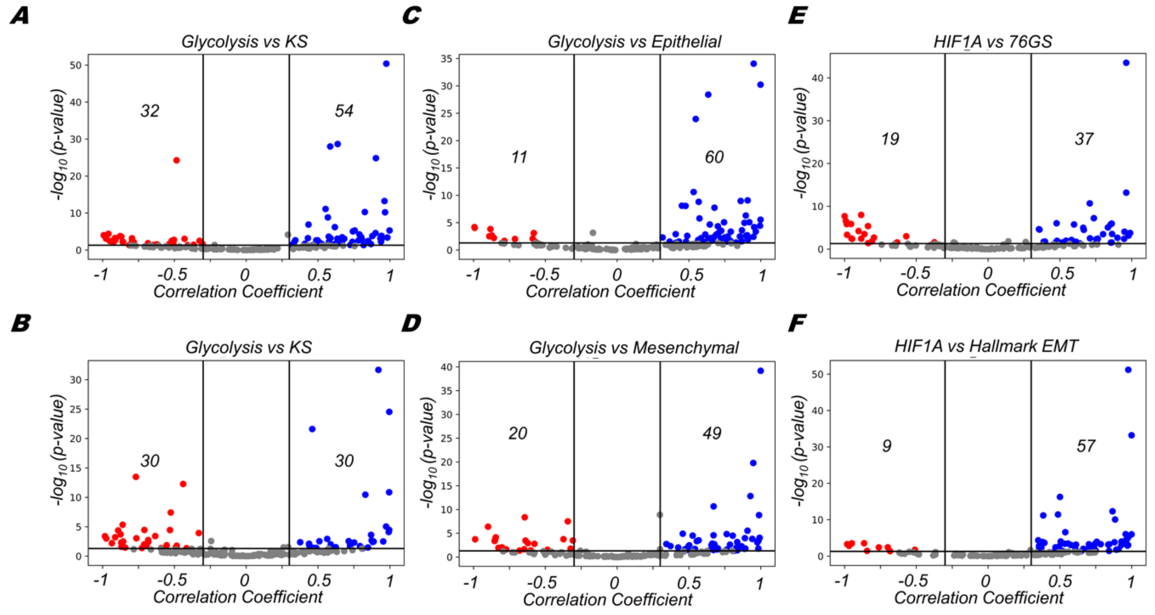

Figure S4. Glycolysis and its regulator HIF1α is more likely to correlate negatively with EMT. Volcano plots depicting the Pearson correlation coefficient and the −log10(p-value) for (**A**) Glycolysis vs KS (**B**) Glycolysis vs 76GS (**C**) Glycolysis vs Epithelial (**D**) Glycolysis vs Mesenchymal (**E**) HIF1α vs 76GS (**F**) HIF1α vs Hallmark EMT. Vertical boundaries are set at correlation coefficient −0.3 and 0.3. Cut-off for p-value is set at 0.05.
